# Microbial Adhesion and Biofilm Formation on Microfiltration Membranes: A Detailed Characterization Using Model Organisms with Increasing Complexity

**DOI:** 10.1155/2013/470867

**Published:** 2013-08-06

**Authors:** L. Vanysacker, C. Denis, P. Declerck, A. Piasecka, I. F. J. Vankelecom

**Affiliations:** ^1^Centre for Surface Chemistry and Catalysis, KU Leuven, Kasteelpark Arenberg 23, P.O. Box 2461, 3001 Heverlee, Belgium; ^2^Laboratory of Aquatic Ecology and Evolutionary Biology, KU Leuven, Charles Deberiotstraat 32, 3000 Leuven, Belgium

## Abstract

Since many years, membrane biofouling has been described as the Achilles heel of membrane fouling. In the present study, an ecological assay was performed using model systems with increasing complexity: a monospecies assay using *Pseudomonas aeruginosa* or *Escherichia coli* separately, a duospecies assay using both microorganisms, and a multispecies assay using activated sludge with or without spiked *P. aeruginosa*. The microbial adhesion and biofilm formation were evaluated in terms of bacterial cell densities, species richness, and bacterial community composition on polyvinyldifluoride, polyethylene, and polysulfone membranes. The data show that biofouling formation was strongly influenced by the kind of microorganism, the interactions between the organisms, and the changes in environmental conditions whereas the membrane effect was less important. The findings obtained in this study suggest that more knowledge in species composition and microbial interactions is needed in order to understand the complex biofouling process. This is the first report describing the microbial interactions with a membrane during the biofouling development.

## 1. Introduction

Bacterial biofilms are ubiquitous in the environment and can be found on almost any hydrated surface. Whilst many of these communities can be used for the synthesis of valuable products and the treatment of waste, others can cause problems in industrial and/or medical applications [1]. Very often in man-made systems, unwanted biofilm formation is referred to as biofouling [2]. Biofouling communities present in wastewater treatment systems, like for example, membrane applications, are extremely complex and contain noncellular material such as minerals, corrosion particles, clay, or silt particles but also, most importantly, numerous bacterial species and other microorganisms [3]. Ranging from membrane bioreactor (MBR) to reverse osmosis systems, the biofouling processes are the same: cells attach to the membrane, start to multiply, produce exopolymeric substances (EPS), and finally block the membrane pores [4]. One of the most important issues of membrane biofouling is the initial attachment of microorganisms, since bacterial attachment is a necessary first step for membrane biofilm formation [5]. Bacterial adhesion by one species on the membrane surface has been extensively studied. However, membrane biofouling as it occurs in purification systems is much more complex as species may interact additionally with each other. For example, it is known that when species are occurring in a biofilm, they behave differently by changing their gene expression and growth rate [6]. They undergo a transition from a planktonic (“loner”) to a community-based existence in which they interact with various bacterial species in close proximity [7]. Some species can coexist and cooperate by exchanging, for example, metabolic products, while they may outcompete each other when nutrients become scarce [8, 9]. From a membrane biofouling point of view, it is important to understand these bacterial interactions. This will not only lead to a complete comprehension of what exactly happens on the membrane surface, but it will also allow to improve filtration processes and fouling control strategies. The present paper reports on the adhesion and biofilm formation of three types of biofoulants on membranes that are ubiquitously used in membrane bioreactors. The objective of this study is to gain insight in to the mutual bacterial interactions and their interactions with the membrane surface. The biofoulants complexity increases stepwise, starting from monospecies *Pseudomonas aeruginosa* over duospecies *P. aeruginosa *and *Escherichia coli* to diluted activated sludge spiked with *P. aeruginosa* and finally activated sludge. 

## 2. Material and Methods

### 2.1. Experimental Design

**Figure 1:**
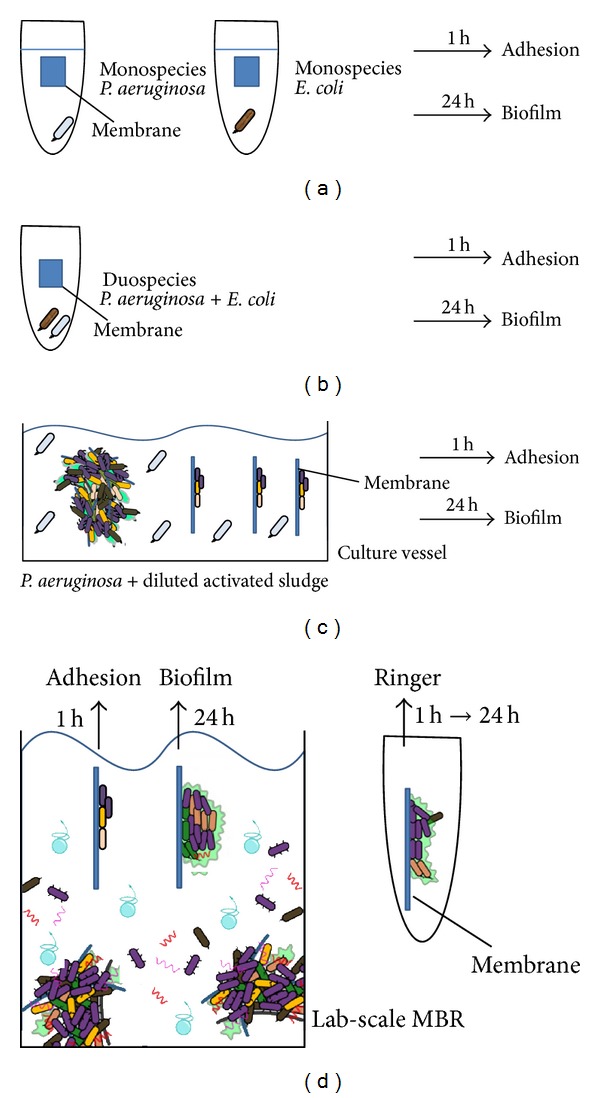
Graphical illustration of the experimental design used in this study. In the first experiment, the adhesion and biofilm formation of monospecies *P. aeruginosa *and *E. coli* were measured on PE, PSF, and PVDF membranes. (a) In a second experiment, the two microorganisms were mixed, and the adhesion and biofilm formation were enumerated. (b) The third experiment was performed in a culture vessel. Diluted activated sludge spiked with *P. aeruginosa *was used as model biofoulants. (c) The fourth experiment used real activated sludge as biofoulant and was done in a lab-scale MBR. In this set up, the microbial adhesion, the biofilm formation in the reactor, and the biofilm formation in Ringer's solution after 1 h adhesion in the reactor were measured (d).

In order to analyze the adhesion and biofilm formation of the microorganisms, four types of static experiments using an increasing biofoulant complexity were performed (Figure 1). At first, a static assay was performed in test tubes using two model monospecies suspensions of *P. aeruginosa *and *E. coli *(Figure 1(a)). Secondly, the two model organisms were mixed in an equal concentration and used as biofoulant in a similar test tube set-up (Figure 1(b)). In a third experiment, the biofouling capacity of diluted activated sludge spiked with *P. aeruginosa*, both in approximately equal concentration,was investigated in a culture vessel (Figure 1(c)). Finally, a fourth experiment was performed in a lab-scale MBR reactor where activated sludge was used as biofoulant (Figure 1(d)). For every experiment, the initial adhesion and the subsequent biofilm formation were evaluated by an incubation time of 1 h and 24 h, respectively. In addition, a third condition was created in the lab-scale MBR assay where the membranes were incubated in Ringer's solution to mimic oligotrophic conditions (Figure 1(d)).

During every experiment three microfiltration membranes, commonly used in MBR systems [10], were tested: polyvinyldifluoride (PVDF), polyethylene (PE), and polysulfone (PSF) membranes. To allow accurate statistical analysis, at least three replicate membranes for every experiment were used. For the mono- and duospecies experiments, *P. aeruginosa *and *E. coli *were quantified using standard plate count. In the third experiment, *P. aeruginosa* was quantified using *P. aeruginosa *gene specific quantitative real-time PCR (qPCR) [11]. In experiment three and four, the activated sludge bacterial community composition was determined by bacterial fingerprints using Automated Ribosomal Intergenic Spacer Analysis (ARISA) [12], and the total amount of cells were quantified by 16S rRNA qPCR [13].

### 2.2. Model Microorganisms and Culture Conditions

As mentioned in the experimental design, *P. aeruginosa, E. coli, *and activated sludge were used as biofoulants. Reasons why *P. aeruginosa *and *E. coli* have been chosen as model organisms are: (1) they are both Gram-negative bacteria, (2) they have approximately the same growth rate and hydrophobicity, (3) they are commonly present throughout aquatic environments [14] and activated sludge [15], and (4) they are the best studied model organisms for biofilm formation since years [16–18]. *P. aeruginosa *(PA14) green fluorescent protein (gfp) labelled (kindly given by Prof. B. Koch, Technical University of Denmark) and *E. coli *(LMG  2092^T^) red fluorescent protein (rfp) labelled (kindly given by Prof. N. Boon, UGent, Belgium) were cultivated in a shaking incubator at 28°C in Lysogeny Broth (LB) (10 g tryptone, 5 g yeast extract, and 5 g NaCl per liter). *P. aeruginosa *cultures were supplemented with 100 mg L^−1^ ampicillin, 10 mg L^−1^ gentamycin, 25 mg L^−1^ kanamycin, and 25 mg L^−1^ streptomycin [19]. *E. coli *was cultured using 50 mg L^−1^ kanamycin and 100 mg L^−1^ nalidixic acid. At the start of each experiment, cells from a 24 h culture were harvested and suspended in sterile Ringer's solution (Oxoid). The initial cell concentration was spectrophotometrically (*λ*_650 nm_) determined, and countable 10-fold dilutions were additionally plated as a control [20].

Activated sludge originating from a lab-scale MBR fed with molasses based wastewater was used. The operational parameters of the MBR and the sludge characteristics are in detail described by Piasecka et al. [21]. During the third experiment (Figure 1(c)), activated sludge was diluted 1000 times and spiked with 10^4^ *P. aeruginosa *cells mL^−1^. 

### 2.3. Membrane Preparation

**Figure 2:**
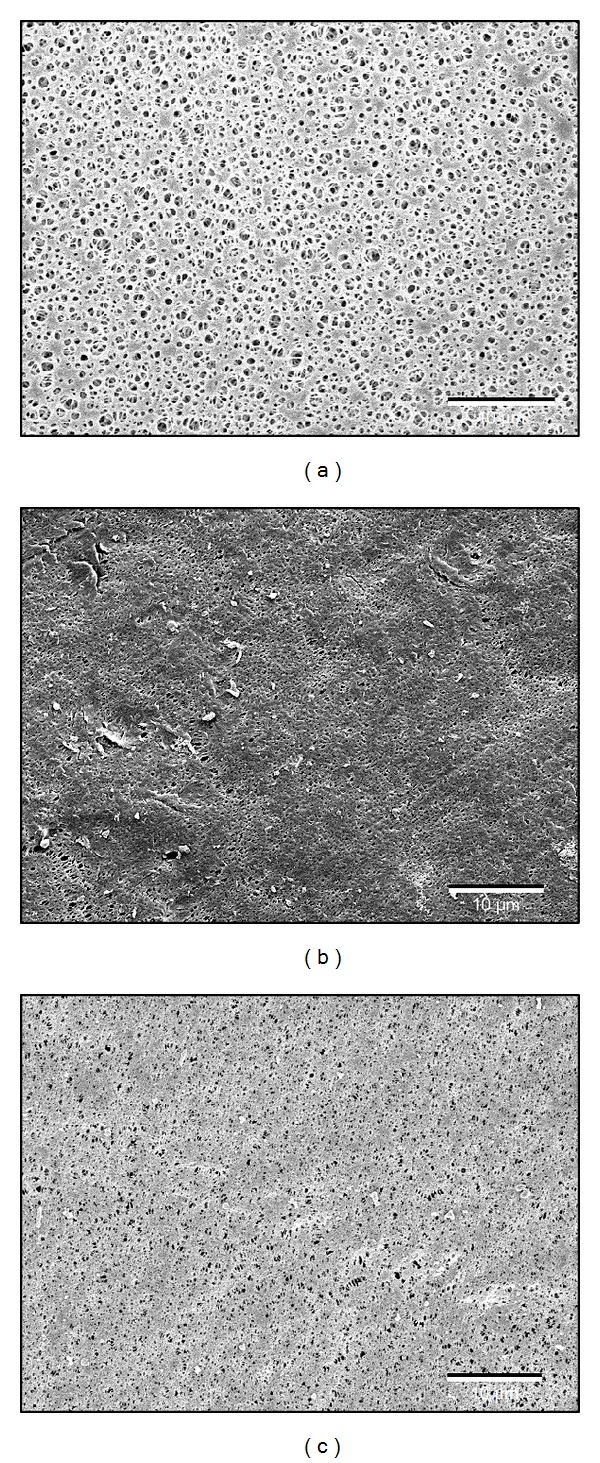
SEM pictures of pristine PE (a), PSF (b), and PVDF (c) membranes. The scale bar represents 10 *μ*m.

Two lab-made and one commercial membrane were used. PVDF and PSF microfiltration membrane sheets were produced via the phase inversion procedure [22–24]. Briefly, a 12 wt% PVDF (Sigma-Aldrich) and 10 wt% PSF (BASF-Ultrason) solution was prepared in dimethylacetamide (Sigma-Aldrich) and N-Methyl-2-pyrrolidone (ACROS) solutions, respectively. Subsequently, the membranes were cast (250 *μ*m thickness) on a polypropylene/polyethylene support (Viledon nonwoven FO 2471, kindly supplied by Freudenberg, Germany). The solvents were allowed to evaporate during 60 s, followed by coagulation of the polymer film in deionized water. The PE membrane was purchased from Kubota (cartridge type 203). These flat sheet membranes were made of chlorinated PE with a nonwoven cloth base. Out of every membrane sheet, replicate coupons of 4.5 cm² were cut. Each coupon was sterilized during three hours in 70% EtOH, followed by two rinsing steps using sterile deionized water to remove the remaining EtOH. Each sterile membrane coupon was stored in a 50 mL falcon tube filled with 40 mL sterile dH_2_O water at 4°C until further use. Each membrane coupon was used only once. The nominal pore size and porosity of the membranes were determined using scanning electron microscopy (SEM, Philips SEM XL30 FEG with Adax dx-4i system) and image processing software (ImageJ) and were compared with data from the literature (Figure 2) [25, 26]. The membrane surface hydrophobicity was determined using contact angle goniometry (VCA Optima video camera system, AST Products, Billerica) with the sessile drop method. The contact angle was measured at least at five different positions and at different time points: immediately after the drop reached the membrane and 10 min later (Table 1).

### 2.4. Experimental Procedure

#### 2.4.1. Bacterial Adhesion and Biofilm Formation of *P. aeruginosa* and *E. coli* under Monospecies and Duospecies Conditions

Membrane pieces of 4.5 cm² were immersed in LB medium containing kanamycin (25 mg L^−1^). After five minutes conditioning, the specific bacterial species was/were added. A cell concentration of 10^8^ cells mL^−1^ of each microorganism was used in the mono- and duospecies experiments [27–29]. To avoid sedimentation of the cells on the membrane, the membranes were placed in a vertical position in small screw capped tubes during 1 h at 20°C on a rotary shaker (4 g). Hereafter, the membranes were removed, washed in sterile Ringer's solution to remove the loosely bound cells, and placed in a new sterile tube [30]. The adhered cells were harvested and quantified, as described in the section “Biofouling community characterization and cell quantification.” In order to observe further biofilm development and since bacterial cells are more likely to attach to surfaces in a low nutrient environment [29, 31], the washed membranes containing adhered cells were incubated for a second time in Ringer's solution to create oligotrophic circumstances. During this step, the membranes were incubated for 24 h at 20°C on a rotary shaker (4 g). After a washing step, the cells were removed from the membrane and quantified as described in section “Biofouling community characterization and cell quantification.”

#### 2.4.2. Adhesion and Biofilm Formation by *P. aeruginosa* Spiked Activated Sludge

The third experiment was performed in a culture vessel with a working volume of 12 L (Nalgene, Thermo Scientific) using activated sludge harvested from a lab-scale MBR [21]. Prior to inoculation, the bacterial density in the biomass was determined in order to have an idea of the number of cells present in the activated sludge. This was done by 16S rRNA qPCR (see section “Biofouling community characterization and cell quantification”). The total cell density was approximately 10^9^ cells mL^−1^. To gradually increase the biofoulant complexity and see the effect of spiked *P. aeruginosa *cells, the sludge was diluted 1000 times using synthetic wastewater (detailed composition is described by Piasecka et al. [21]). The cells were allowed to adapt and acclimatize to their new environment by mixing the suspension during 24 h. Thereafter, the 10^5^ cells mL^−1^ sludge cells were spiked with approximately 10^4^ *P. aeruginosa *cells mL^−1^ and mixed during 10 min, and finally the membrane pieces were placed in the culture vessel. After 1 h and 24 h incubation, the adhesion and biofilm formation were, respectively, enumerated as described in section “Biofouling community characterization and cell quantification.”

#### 2.4.3. Adhesion and Biofilm Formation by Activated Sludge in a Lab-Scale MBR

In the fourth experiment, the attachment and biofilm formation of microorganisms present in activated sludge were investigated (Figure 1(d)). A description of the lab-scale MBR can be found in Bilad et al. [32] and on http://www.biw.kuleuven.be/cok/membrane2/. The operational parameters and wastewater characteristics are mentioned by Piasecka et al. [21]. Three replicates of each membrane type were randomly immersed in the reactor tank. The membranes were incubated during 1 h in order to measure the cell adhesion. After a washing step in Ringer's solution, the cells on the membranes were removed (see below), the total amount of cells was quantified, and bacterial community fingerprints were generated, as described in the following section. In addition, two biofilm formation assays with different environmental conditions were performed: (1) after 1 h incubation in the reactor, the washed membranes were further incubated in sterile Ringer's solution (oligotrophic biofilm condition) since bacterial cells are more likely to attach to surfaces in a low nutrient environment [29, 31] and (2) the membranes were further incubated during 24 h in the reactor itself to compare the biofilm formation with the oligotrophic biofilm condition (Figure 1(d)).

#### 2.4.4. Biofouling Community Characterization and Cell Quantification

The biofouling was harvested in the same way for the four experiments and for the adhesion and biofilm formation (Figure 1). Briefly, cells were removed from the membrane by scraping using a cell scraper, sonication (10 min, 42 kHz ± 6%, Bransonic 2510), and vortex (30 s, max speed) in 10 mL Ringer's solution [33]. Biofouling associated with *P. aeruginosa *and *E. coli *were quantified by the standard plating method using LB medium with the required antibiotics. Activated sludge biofouling was characterized using DNA-based assays. For this end, DNA of the acquired cell suspension was extracted using the Mobio Ultraclean soil DNA kit (Cambio) [34]. Afterwards, the total amount of cells was quantified by 16S rRNA qPCR [13], and bacterial community fingerprints were generated by ARISA [12]. The spiked *P. aeruginosa *in activated sludge was measured using a species specific qPCR, as described by Shannon et al. [11]. 

#### 2.4.5. Data Analysis

Depending on the experimental set-up, different statistical analyses were performed. By one-way analyses of variance (ANOVA), membrane effects were tested on the bacterial cell densities for each species and condition separately. By two-way ANOVA, the amount of extracted cells (expressed as bacterial cell density) and bacterial species richness (total number of Operational Taxonomic Units (OTU) detected by ARISA) were further analyzed with experimental condition (mono- versus duospecies (Figures 1(a) and 1(b)); adhesion versus biofilm versus Ringer (Figure 1(d))) and membrane type as explanatory variables. Bacterial cell densities were logarithmically transformed prior to analysis. Being mainly interested in the interaction between species and membranes, the significant main effects were further explored by comparing all combinations using *post hoc* Tukey honest significant difference tests. All univariate analyses were performed with Statistica 11.0 software.

Concerning the bacterial community data analysis, the associations among the community composition (height data of OTUs) were explored firstly using Principal Component Analyses (PCA). Secondly, the relationship between the observed bacterial community composition (height data of OTUs) and the experimental factors (membrane type and experimental condition (adhesion versus biofilm (Figure 1(c))); adhesion versus biofilm versus Ringer (Figure 1(d))) was explored by means of Redundancy Analysis (RDA). RDA is a direct gradient ordination technique that allows to formally test for the significance of experimental factors on community data, as well as the interaction effects among factors [35]. Significance tests were performed with random Monte Carlo permutation tests (9999 unrestricted permutations per test). Both analyses were performed using the software package Canoco for Windows, version 4.5 (Biometris Plant Research International).

## 3. Results

### 3.1. *P. aeruginosa *and *E. coli*: Monospecies Condition

**Figure 3:**
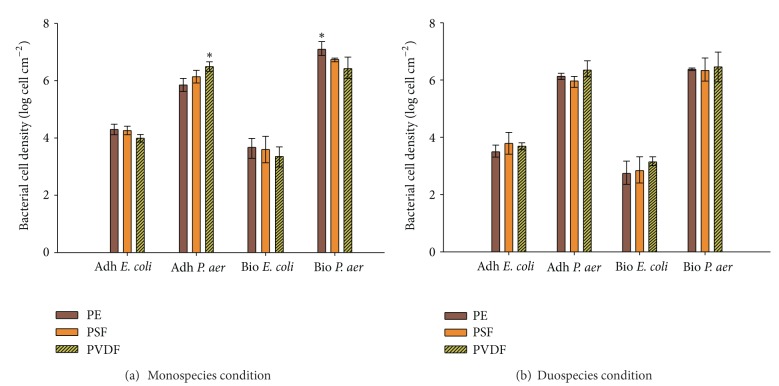
Bacterial cell density expressed in log cells cm^−2^ membrane after adhesion and biofilm formation of *P. aeruginosa *and *E. coli *in monospecies (a) and duospecies (b) conditions on PE, PSF, and PVDF membranes. Cells were enumerated using standard plating and log transformed before analysis. Error bars represent standard deviation (*n* = 3). *P. aer* = *P. aeruginosa*, Adh = adhesion, Bio = Biofilm, and * = significantly different.

Using a static assay in test tubes, the adhesion rate and biofilm formation of *P. aeruginosa *and *E. coli *were measured (Figure 1(a)). The bacterial cell densities are shown in Figure 3(a). No membrane effect was found for *E. coli *during the adhesion (one-way ANOVA, *P* = 0.099) and biofilm formation (one-way ANOVA, *P* = 0.636). On the other hand, a small but significant membrane effect was found during the *P. aeruginosa* adhesion (one-way ANOVA, *P* = 0.024). A better attachment was detected on the PVDF membrane in comparison with the PE membrane (*post hoc* Tukey test, *P* = 0.020, Figure 3(a)).

The cell increase during the biofilm formation was rather low for both microorganisms. *P. aeruginosa *showed a small increase in cells, especially on the PE membrane. On the other hand, *E. coli *was not able to produce a biofilm as lower cell densities were found in comparison with the adhesion. When the two model organisms are mutually compared, *P. aeruginosa *adhered approximately 100 times more than *E. coli* on each membrane type (Figure 3(a)).

### 3.2. *P. aeruginosa *and *E. coli*: Duospecies Condition

In the second experiment (Figure 1(b)), the adhesion and biofilm development was examined in a duospecies condition. The “coexistence effect” was analyzed by two-way ANOVA with coexistence and membrane type as explanatory variables. For the coexistence variables, the values of the cell densities measured during the mono- and duospecies experiments were used.

 A significant coexistence effect was found during the *E. coli* attachment (Table 2, *P* < 0.001), meaning that *E. coli *was less able to bind to the membranes when *P. aeruginosa *was present. On the other hand, the adhesion of *P. aeruginosa *was not limited when *E. coli *was present as no significant coexistence effect was found (*P* = 0.74). During the adhesion step, a membrane effect was found for *P. aeruginosa* (Table 2, *P* = 0.005). A *post hoc* Tukey test showed that this effect was mainly attributed to the differences between PE and PVDF in the monospecies experiment (*P* = 0.02), which has been mentioned in the previous section. Different significant combinations were found for *E. coli *(statistical data not shown), but as no main membrane effect was found (Table 2, *P* = 0.39), these differences are attributed to the strong coexistence effect (mono versus duospecies).

When looking at the biofilm formation (Figure 3(b)), a *P. aeruginosa *coexistence effect showed up (Table 2, *P* = 0.03), meaning that, in contrast with the attachment, the biofilm formation of *P. aeruginosa *is affected by the presence of *E. coli*. Also the other way around, the biofilm formation of *E. coli *was still hampered when *P. aeruginosa *was present. For both species, no pairwise combination effects were found (statistical data not shown). This underlines the poor membrane effect.

### 3.3. Activated Sludge Spiked with *P. aeruginosa*

**Figure 4:**
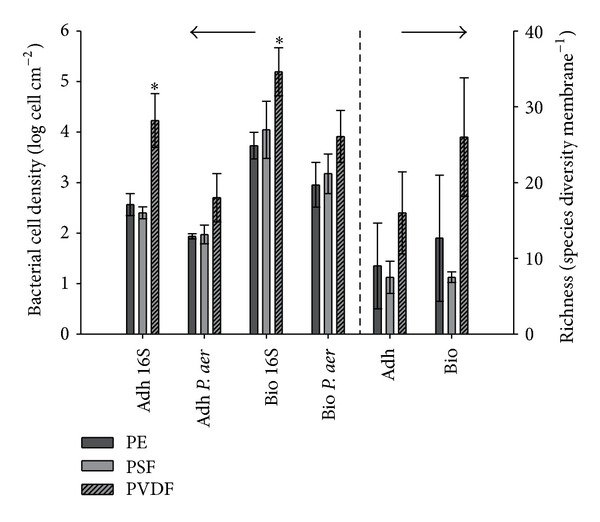
Bacterial cell density (left axis) and species richness (right axis) expressed, respectively, as log cells and species diversity cm^−^² membrane when diluted activated sludge spiked with *P. aeruginosa *was used as biofoulant. The bacterial cell density was enumerated with 16S and *P. aeruginosa *specific qPCR. The species richness was calculated as the sum of the number of peaks (OTU's) within each ARISA electropherogram. Error bars represent standard deviation (*n* = 3). *P. aer* = *P. aeruginosa*, Adh = adhesion, Bio = Biofilm, and * = significantly different.

To gradually increase the biofoulant complexity, activated sludge was diluted to approximately 10^5^ cells mL^−1^ and spiked with 10^4^ *P. aeruginosa *cells mL^−1^ (Figure 1(c)). To have an idea how many bacterial cells (16S rRNA) were attached in total and what the proportion of *P. aeruginosa *was within the activated sludge community on each membrane, both cell types were quantified after 1 and 24 h using qPCR (Figure 4). The bacterial cell density in the culture vessel after 24 h filtration was 7.70 ± 2.29 × 10^3^ *P. aeruginosa *cells mL^−1^ and 4.29 ± 0.78 × 10^7^ total bacteria cells mL^−1^.

Two-way ANOVA models were performed on the adhesion and biofilm data separately using membrane and organism (*P. aeruginosa *and 16S cell) type as variables. All the variables were significantly different (Table 3). During the adhesion and the biofilm formation, high cell densities were found on the PVDF membranes especially when the universal 16S primers were used (Figure 4). 

*P. aeruginosa *was well represented on the membrane surface in comparison to the other community members. Twenty-one and 38% of the microbial population were *P. aeruginosa *during the adhesion process on the PE and PSF membrane, respectively. Also during the biofilm formation, *P. aeruginosa *was able to attach to the PE and PSF membrane but in smaller quantities: 16 and 13% of the population were assigned as *P. aeruginosa, *respectively. The 16S cell densities were much higher on the PVDF. This led to a lower *P. aeruginosa *proportion: 2.3 and 5.3% during the adhesion and biofilm formation, respectively (Figure 4).

Similar, but less pronounced, results were found using the species richness (Figure 4). The species richness in the culture vessel was measured as 35 ± 3.9 OTUs mL^−1^. The PVDF membrane had for the biofilm condition a high species richness and was significantly different from the PSF membrane (statistical data not shown). No clear differences were found between the adhesion and biofilm species richness data on the same membrane. 

**Figure 5:**
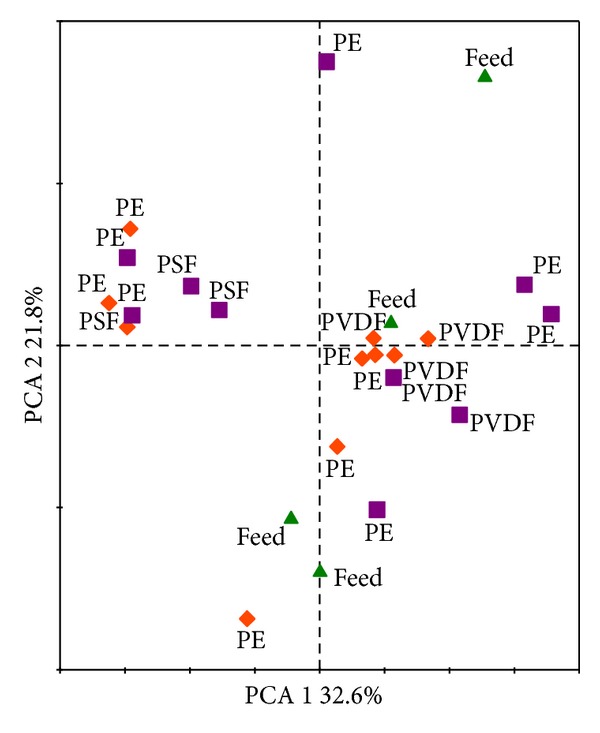
PCA ordination diagram illustrating the relationships among the bacterial communities found on the PE, PSF, and PVDF membranes. The diagram is based in the height data of the ARISA data. Samples corresponding to the adhesion data are represented by squares and samples from the biofilm data by diamonds. The bacterial community in the culture vessel (feed) is plotted as triangles. The diagram shows the first and second PCA axes, that, respectively, account for 32.6% and 21.8% of the explained variability.

In addition to the species richness, bacterial community profiles were generated using the ARISA dataset and were analyzed and visualized by RDA and PCA, respectively. In contrast with the results found using the species richness, no membrane effect was found (*P* = 0.08). Also the adhesion and biofilm communities were similar (*P* = 0.294). These results are also visible on the PCA plot (Figure 5), where the adhesion and biofilm samples are randomly spread. Although the membrane effect was not significant, most of the PVDF membrane samples have the tendency to group together. These profiles might explain the significant effects found using the bacterial density and species richness data.

### 3.4. Activate Sludge in a Lab-Scale MBR

**Figure 6:**
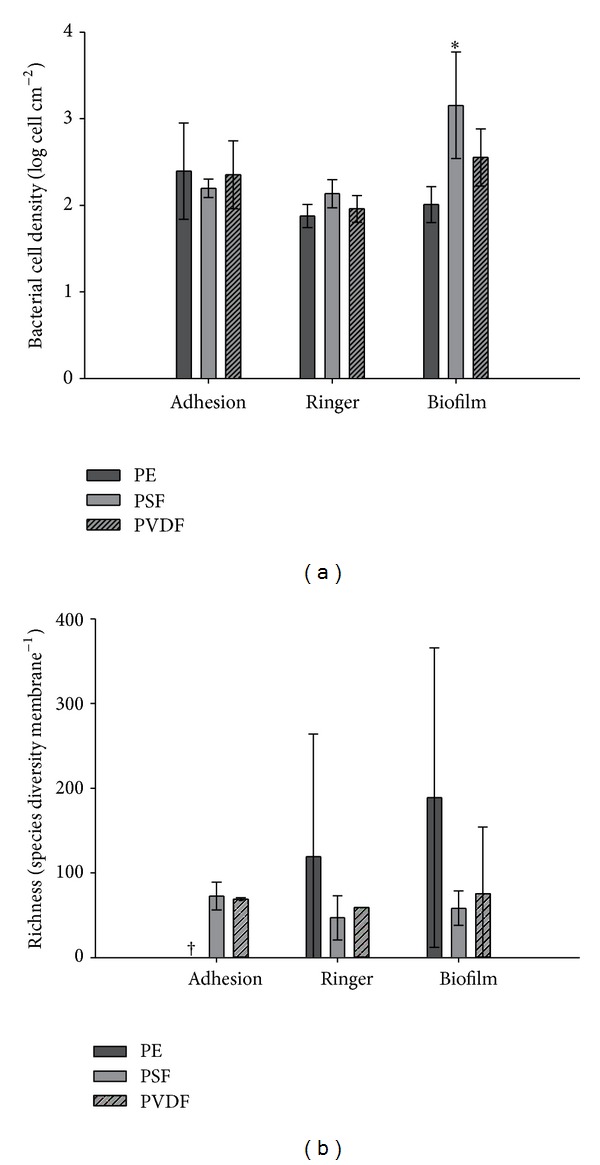
Bacterial cell density (a) and species richness (b) expressed, respectively, as log cells and species diversity cm^−^² membrane after adhesion and biofilm formation of activated sludge on PE, PSF, and PVDF membranes. Bacterial cell densities were enumerated using 16S rRNA qPCR and log transformed before analysis. The richness was calculated by the sum of the number of peaks (OTU's) within each ARISA electropherogram. Results for the adhesion on the PE membrane are not available due to repeated unsuccessful PCR amplification (^†^). Error bars represent standard deviation (*n* = 3). Adhesion = 1 h incubation in the lab-scale MBR, Ringer = 1 h incubation in the lab-scale MBR followed by 24 h biofilm in Ringer's solution, biofilm = 24 h incubation in the lab-scale MBR, and * = significantly different.

The results of the total amount of cells, quantified by 16S rRNA qPCR, are shown in Figure 6(a). The data was further analyzed with a two-way ANOVA. A significant interaction (*P* = 0.047) was observed (Table 4), which could be assigned to the high cell densities found in the PSF-biofilm condition (Figure 6). These results are highlighted by the *post hoc* Tukey test in Table 5. No analogue results were found during the adhesion or biofilm formation in Ringers' solution on the other PE membranes (Figure 6).

The ARISA dataset was used to compare the overall community structure among samples. The generated electropherograms delivered OTUs ranging from 224 to 1148 bp. The majority of them had a length between 243 and 1086 bp. The species richness, which is the sum of the OTUs in each sample, is shown in Figure 5 (right). Due to repeated unsuccessful PCR amplification, PE-adhesion data were not available, and no statistical tests using the species richness were performed. A high variation inside each treatment (adhesion, Ringer, biofilm) and each membrane was found (Figure 5). Also the samples processed after 1 h adhesion and 24 h biofilm formation in the MBR showed a much higher species diversity compared to the Ringer treatment.

**Figure 7:**
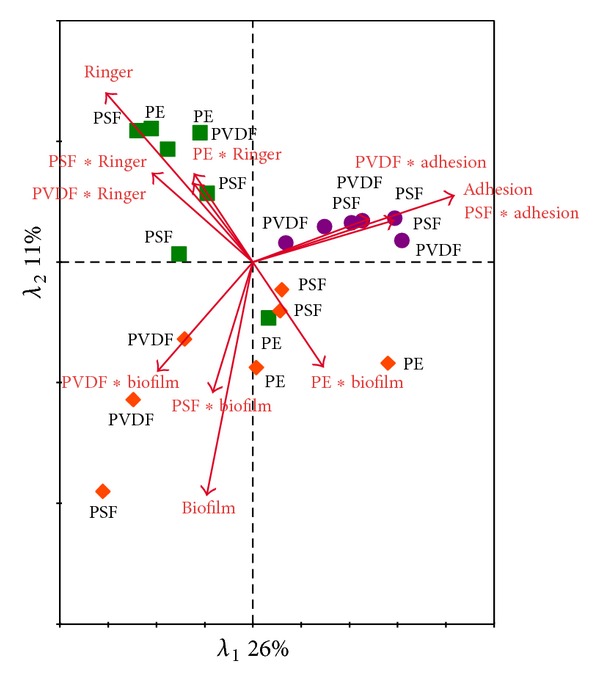
RDA ordination diagram based on the bacterial ARISA data, representing the interaction between membrane type and treatment (B). *λ*_1_ represents the eigenvalue axis 1; *λ*_2_ represents the eigenvalue axis 2. Arrows represent significant environmental variables superimposed onto the ordination; the length of the arrow indicates the correlation between the environmental variable and the ordination axis. Adhesion data (dots): 1 h incubation in the lab-scale MBR, Ringer data (squares): 1 h incubation in the lab-scale MBR followed by 24 h biofilm in Ringer's solution, and biofilm data (diamonds): 24 h incubation in the lab-scale MBR.

Using RDA, the effects of membrane and treatment (adhesion, Ringer and biofilm) type on the bacterial community profiles were explored. Similar as with the cell densities, RDA showed a significant treatment and interaction effects (membrane∗treatment) (Table 5). The treatment effect explained 36% of the total bacterial community variation compared with only 8% for the, non significant, membrane effect. Moreover, the interaction effect was more pronounced than the main effects of the experimental factors, as it explained 43% of the community variability. These results are also visible in Figure 7. The different conditions have the tendency to group together whereas the membrane types are more randomly dispersed in the community profile.

## 4. Discussion

### 4.1. *P. aeruginosa *and *E. coli*: Monospecies Condition

Initial bacterial adhesion to abiotic surfaces is likely to be highly dependent on physicochemical and electrostatic interactions between the bacterial envelope itself and the substrate, which is often conditioned by the fluids to which it is exposed [36]. Generally, for aqueous applications, fouling is less pronounced on hydrophilic membranes than on hydrophobic membranes [29, 37, 38]. The PE membrane had initially a high hydrophobicity, but the contact angle changes rapidly in time as water penetrated inside the membrane pores during the measurement. This phenomenon occurred also on the PVDF membrane. The PSF membrane had the highest stability (Table 1). Normally, a higher affinity was expected for the PE and PSF membranes. This was not the case, since hardly any membrane effect could be observed. Only during *P. aeruginosa* adhesion, a slightly higher affinity was found for the PVDF membrane in comparison to the PE membrane (Figure 3(a)), but this membrane effect disappeared after 24 h of further incubation, and a higher *P. aeruginosa* number were then detected on the PE membrane. 

### 4.2. *P. aeruginosa *and *E. coli*: Duospecies Condition

Once microorganisms are strongly attached to the membrane, and after the EPS production is activated, the microorganisms eventually become embedded in the hydrated matrix and are immobilized. As a result, the cells are influenced by the exchange of nutrients with neighboring cells in the biofilm. An important feature is that the microorganisms are immobilized in relatively close proximity to one another, becoming continuously confronted with dynamic changes. Therefore, the success rate of any given microorganism in a multispecies biofilm strongly depends on the behavior of the other participants [39]. A first factor determining the fitness of a bacterial strain with respect to a given set of nutrients is its growth rate [40]. Although the growth rates of our two model organisms, at least in monoculture (approximately 26 and 31 min for *P. aeruginosa *and *E. coli,* resp.), were similar, a higher attachment for *P. aeruginosa *was found. Approximately 100 and 1000 times more *P. aeruginosa *cells were quantified in the adhesion (Figure 3(a)) and biofilm (Figure 3(b)) assay, respectively. A second possible reason is the difference in motility. Indeed, the ability to attach or to detach from a surface and the possibility to migrate over the substrate are features affecting the biofouling potential of species. *P. aeruginosa *and *E. coli* possess polar monotrichous and peritrichous flagellation, respectively, allowing swarming/swimming motility [41]. However, compared to the peritrichous flagella observed in *E. coli*, the polar flagellum in *P. aeruginosa* is driven by sodium motive force and may then propel the bacterium at relatively high speeds towards the membrane. In contrast, *E. coli* cells, propelled by numerous proton-powered lateral flagella, seem to be effective for attachment and biofilm formation [42] but possibly not powerful enough in comparison with the motility of *P. aeruginosa*. Another distinct type of motility, named twitching, is mediated by type IV pili located at one or both poles of the cell [41]. Although some pathogenic *E. coli *strains contain a related type IV motility system [43], twitching motility did not occur in the LMG strain used here. The differences in motility between the two species may explain the adhesion differences found in Figure 3. Also the fact that no condition effect (Table 2) was found for *P. aeruginosa *during the adhesion suggests that *P. aeruginosa *can outcompete the attachment of *E. coli*. 

Interesting are the differences found between adhesion and biofilm formation. Here, a decrease in *E. coli *cells after 24 h incubation in oligotrophic circumstances was found, which is in contrast with the increase of *P. aeruginosa* in monospecies condition (Figure 3(a)). This suggests that, although *E. coli *could attach to the membranes, the bacterium was not able to stay on it and produce a biofilm. This is quite surprising as biofilm formation is normally enhanced by environmental stress in many bacterial species and seems to be promoted by nonoptimal growth conditions or even by cellular stresses [44], here mimicked by the oligotrophic Ringer's solution. The decrease in *E. coli* cells was even more pronounced when both species were mixed (Figure 3(b), Table 2 coexistence effect), suggesting that rather antagonistic effects occur between the two species. These findings are in contrast with the findings of Liu and Li [45], where an enhanced attachment of *E. coli *on a *P. aeruginosa *biofilm layer was shown on porous media in LB broth. Also it is suggested that during the biofilm maturation, interbacterial adhesion, rather than direct contact with the substrate, leads to progressive buildup of the mature biofilm. On the other hand, it is also known that some species produce inhibitor substances against others [46]. Because the production of antimicrobial compounds can be a metabolic burden on the cell, some species evolved to start the production of such components when their cell numbers are high enough to ensure that the compound will reach an effective concentration, a phenomenon known as quorum sensing [39]. Different quorum sensing dependent systems have been described between *E. coli* and* P. aeruginosa *[47, 48]. As a different pattern was found between the adhesion and biofilm assay, it is conceivable that quorum sensing is one of the occurring mechanisms, but further experiments are required to confirm these hypotheses. However, the more effective colonization behavior of *P. aeruginosa *on every membrane type was clearly highlighted in these experiments.

### 4.3. Activated Sludge Spiked with *P. aeruginosa*

To gradually increase the complexity of the biofoulants, activated sludge harvested from a lab-scale MBR was diluted and spiked with *P. aeruginosa*. It is well known that activated sludge contains a very complex community, and many different factors affect the exact community composition [49]. Different species are present, and each group of species has its own habitat and niche. Examples are floc-forming, filamentous, nitrifying, and organotrophic bacteria. By introducing a new habitat, like a membrane, some species can bind to it and exhibit a preferred membrane associated life form. This is probably the reason why the microbial communities on membrane surfaces are very different from the ones in the suspended biomass [21, 50–59].

In the presented experiment, a quite important membrane effect was found (Table 3). The PVDF membrane, which did not have a specifically high biofouling tendency in the mono-, duospecies, or lab-scale MBR experiments, showed high cell densities and species richness (Figure 4). As this phenomenon was observed during the adhesion and the biofilm formation, this may suggest that some species present in the culture vessel had a high affinity for this polymer. Although no significant membrane effect was found using RDA, the PVDF samples had the tendency to cluster together in the PCA plot (Figure 5). This suggests that a slightly different community was present on the PVDF membrane. Quite interesting is that at the time this experiment was performed, the lab-scale MBR, where the sludge was harvested from, was running using only PVDF membranes. Perhaps some species developed the ability to bind on this polymer and formed a biofilm on it, and maybe this can explain why a higher species richness was found in comparison with PSF and PE. During the fourth experiment, which was performed in the lab-scale MBR itself (Figure 1(d), discussed in the following section), the reactor was running without membrane, and this might be a reason why no clear differences in cell densities and high variation in species richness were found among the membranes (Figure 6). Or maybe cells only need to adhere when they are in a stressful environment (the culture vessel in comparison with the MBR reactor), like in most biofilm formation. Adaptation to environmental stress is indeed well documented for the used model organisms. Boles et al. demonstrated that *P. aeruginosa* undergoes genetic diversification during short-term biofilm growth, which might allow increased resistance of the population to environmental stresses [60]. 

Compared to the mono- and duospecies experiments, it seems that attachment of *P. aeruginosa *was not really influenced by other community members (Figure 3 versus Figure 4). In brief, if one analyses Figure 3 in more detail, one can see that for both the mono- and duospecies experiments, with an inoculation of 10^8^ cells mL^−1^, the enumerations of *P. aeruginosa *during the adhesion and biofilm formation are quite similar in terms of order of magnitude (adhesion 10^6^ cells cm^−1^; biofilm 10^7^ cells cm^−1^). Next, if one analyses Figure 4, one can see that for activated sludge spiked with *P. aeruginosa*, with an inoculation of 10^4^ *P. aeruginosa *cells mL^−1^, the adhesion and biofilm formation are 10^2^ and 10^3^ cells mL^−1^, respectively. This means that, with respect to the original number of inoculated cells, the cell attachment on the membrane is independent from the biofoulant complexity, as the difference of the cell attachment is identical to the difference between the number of inoculated cells, namely, 10^4^. This means that *P. aeruginosa*, even though the bacterium is in competition with other community members, still can preserve its place on the membrane. This is very clear for the PSF and PE membranes and highlights again the good attachment and biofilm properties of the bacterium (Figure 4). The reason why *P. aeruginosa *is such an excellent biofilm pioneer is probably due to its high adaptive response to environmental stress. It was recently reviewed that 303 genes are differentially expressed under different conditions [61]. Functional categorization of the genes revealed that the most highly differentially regulated genes encode membrane proteins, followed by genes involved in the transport of small molecules (such as QS molecules), adaptation and protection, and transcriptional regulators, indicating an adaptive response of *P. aeruginosa* to the changing environment [61].

### 4.4. Activate Sludge in a Lab-Scale MBR

In order to mimic a more representative biofouling formation, membrane pieces were submerged in a lab-scale MBR and processed in three different ways (1 h adhesion, 1 h adhesion followed by 24 h further incubation in Ringer, or in the MBR). When both the bacterial density (Table 4) as well as the community composition (Table 5) was used, a strong main treatment and interaction (membrane ∗ treatment) effect was found. Although the membranes were incubated 24 h longer in the reactor or in Ringer's solution, the differences with the cell densities after 1 h adhesion were rather small. This suggests that in 1 h time, most of the membrane surface was covered (Figure 6). Only PSF-biofilm showed a higher enumeration during the MBR biofilm formation and strongly influenced the *post hoc* Tukey test (Table 4). The reason why is not clear as the effect is not present in the other assays. Notwithstanding the fact that the membrane replicates were randomly located in the reactor, small differences in aeration and therefore a lower membrane scouring cannot be neglected at lab-scale. Concerning the species richness, high variations were found between the membranes. Also the species diversity in the 24 h Ringer treatment was much lower compared to the 1 h adhesion and 24 h biofilm formation in the reactor. This suggests that some species could not stay in the freshly formed biofilm (decrease in diversity (Figure 6(b)), while other species use the free space on the membrane and start to proliferate (unchanged density (Figure 6(a)). Also this confirms why significantly different community patterns were found using RDA for the three treatments (Table 5). The RDA plot illustrates that the samples had the tendency to cluster together along the treatments (adhesion, Ringer, and biofilm) rather than along the different membrane types (Figure 7).

## 5. Conclusion

The bacterial attachment and biofilm formation on three types of polymeric microfiltration membranes were evaluated in this study using different static assays. Notwithstanding the fact that membranes with quite different characteristics (PE, PSF, and PVDF) were chosen, the membrane effects in the four experiments were either nonexistent, rather low, disappearing after longer incubation times, or nonrepetitive when different biofoulants were used. On the other hand, coexistence or treatment effects were significant in most of the cases. This means that the membrane biofouling was most influenced by the species composition, how species interact with each other, and the environmental situations in which the membranes were situated. As expected, when more complex but rather realistic biofoulants were used, the biofouling behavior gets more difficult to predict. For example, it is clear that when one dominant species, such as *P. aeruginosa*, with good attachment capabilities is present, high fouling tendencies are expected on every membrane by this particular species. On opposite sides, when a feed is mainly composed by *E. coli *like species, which are less able to attach to a substrate, the biofouling formation will be slower (experiments 1 and 2). However, when other species are present together with *P. aeruginosa* like members, but they are still in minority, changes in biofouling pattern may occur. In this study, this was highlighted by the high enumeration on the PVDF membrane in experiment 3. Moreover it was suggested that not only the species diversity, but also the origin of the community had an important effect on the biofouling tendencies. Similar ecological assays may lead to the identification of microbial key players and provide many exciting insights into biofouling related microorganisms.

## Figures and Tables

**Table 1:** Characteristics of the membranes used in this study.

	Type	Pore size (*μ*m)	Surface porosity (%)	Contact angle (°)	
				5 sec	10 min
PSF	Labmade	a	a	74 ± 1	71 ± 2
PVDF	Labmade	0.01	19	73 ± 7	49 ± 2
PE	Kubota	0.4	25	103 ± 2	73 ± 3

^a^Not possible to determine with ImageJ.

**Table 2:** Two-way factorial ANOVA results of the duospecies experiment using *P. aeruginosa *and *E. coli*. Bacterial cell density (log cells cm^−2^ membrane) was selected as dependent variable and membrane type (PSF, PVDF, and PS) and bacterial coexistence (mono versus duospecies) as predictor variables.

	*F*	*P*
Duospecies experiment—adhesion		
*E. coli *		
Membrane	1.004	0.391
Coexistence (mono- and duospecies)	26.63	**<0.001**
Membrane ∗ coexistence	2.083	0.162
*P. aeruginosa *		
Membrane	8.50	**0.005**
Coexistence (mono- and duospecies)	0.11	0.744
Membrane ∗ coexistence	2.11	0.164
Duospecies experiment—biofilm		
*E. coli *		
Membrane	10.03	0.967
Coexistence (mono- and duospecies)	11.41	**0.005**
Membrane ∗ coexistence	1.513	0.263
*P. aeruginosa *		
Membrane	1.274	0.321
Coexistence (mono- and duospecies)	5.639	**0.032**
Membrane ∗ coexistence	1.937	0.198

Significant values are in bold. Bacterial cell density data were log transformed before analysis.

**Table 3:** Two-way ANOVA results of the activated sludge experiment spiked with *P. aeruginosa *in the culture vessel. Bacterial cell density (log cells cm^−2^ membrane) was selected as dependent variable and membrane (PSF, PVDF, and PS) and organism (*P. aeruginosa* and 16S) as predictor variables.

	*F*	*P*
Spiked activated sludge experiment—adhesion		
Membrane	38.77	**<0.001**
Organism (*P. aeruginosa* and 16S)	39.26	**<0.001**
Membrane ∗ organism	6.413	**0.007**
Spiked activated sludge experiment—biofilm		
Membrane	31.67	**<0.001**
Organism (*P. aeruginosa* and 16S)	6.532	**0.016**
Membrane ∗ organism	11.96	**<0.001**

Significant values are in bold. Bacterial cell density data were log transformed before analysis.

**Table 4:** Two-way factorial ANOVA and *post hoc* Tukey results of the activated sludge experiment in the lab-scale MBR. Bacterial cell density (log cells cm^−2^ membrane) was selected as dependent variable and membrane type (PSF, PVDF, and PS) and treatment (adhesion, biofilm formation in Ringer's solution, and biofilm formation in lab-scale MBR) as predictor variables. The pairwise comparison was performed between the bacterial cell density (log cells cm^−2^ membrane) among the different membrane types and treatment. Bacterial cell density data were log transformed before analysis.

Activated sludge experiment in lab-scale MBR	*F*	*P*
Membrane	3.030	0.073
Treatment (adhesion-Ringer-biofilm)	6.419	0.008
Membrane ∗ treatment	2.972	0.047
*Post hoc* Tukey test		
PSF biofilm-PE biofilm		0.016
PSF biofilm-PE Ringer		0.006
PSF biofilm-PSF Ringer		0.034
PSF biofilm-PVDF Ringer		0.011

**Table 5:** Results of RDA permutation analyses on bacterial community data derived from ARISA, testing for the effects of membrane type (PE, PSF, and PVDF), treatment (adhesion, Ringer, biofilm), and the interaction between both factors. *λ*_1_ and *λ*_2_ represent the eigenvalues of the first and second axes. *λ*_tot_ represents the percentage of total community variation explained by the experimental factors.

	*λ*_1_	*λ*_2_	*λ*_tot_	*F*	*P*
Static assay in lab-scale MBR					
Membrane	0.066	0.014	8%	1.062	0.382
Treatment (adhesion-Ringer-biofilm)	0.247	0.113	36%	4.809	**<0.001**
Membrane ∗ treatments	0.263	0.114	43%	2.161	**0.007**

Significant values are in bold.
